# The gap in medical education: the missing element of digital health economics education - a prospective cross-sectional pilot study

**DOI:** 10.3389/fmed.2025.1624053

**Published:** 2025-09-25

**Authors:** Stefan Hertling, Franziska Loos, Fabrizio Bambina, Oliver Schöffski, Isabel Graul, Ekkehard Schleussner

**Affiliations:** ^1^Department of Obstetrics and Gynecology, University Hospital Jena, Jena, Germany; ^2^Practice for Orthopedics and Shoulder Surgery Leipzig, Leipzig, Germany; ^3^Asklepios Orthopedic Clinic Lindenlohe, Specialist Clinic for Orthopaedics and Spinal Surgery, Schwandorf, Germany; ^4^Health Economics, Friedrich-Alexander University of Erlangen-Nuremberg, Nuremberg, Germany; ^5^Department of Trauma, Hand and Reconstructive Surgery, University Hospital Jena, Jena, Germany

**Keywords:** health economics, medical teaching, students, faculties of medicine, medical education, pilot study, digital health economics education

## Abstract

**Background:**

The increasing economization of healthcare requires greater integration of health economics content into medical education.

**Objective:**

This study examines medical students’ attitudes toward health economics and their preferred teaching formats.

**Materials and methods:**

This prospective cross-sectional pilot study is based on a survey of medical students at the 36 medical faculties in Germany between April and October 2021. For this purpose, questionnaires were established. We received feedback from the participating students through two 24-point questionnaires.

**Results:**

3042 students of human medicine took part. The results reveal a significant gap in education and care: 75% of respondents consider their knowledge to be insufficient, while 93% are in favor of digital teaching formats such as e-learning. Significant differences in approval are evident according to age and stage of study, but not according to gender. Based on these results, the concept of digital health economics education (DHEE) is proposed as a forward-looking teaching concept that conveys health economics content digitally, interdisciplinarity and in a way that is firmly anchored in the curriculum. This form of learning offers great potential for addressing structural challenges such as resource scarcity, increasing economic pressure to make decisions and a lack of management skills in everyday medical practice. Close cooperation between medicine and health economics in research and teaching is required to enable sustainable integration into the curriculum.

## Introduction

The Hippocrati Oath obliges doctors to consider the well-being of their patients in all medical decisions and to avoid causing harm. To meet this ethical standard in an increasingly complex healthcare system, in-depth knowledge of health economics is required in addition to professional expertise (1). In view of limited resources, growing financial burdens, and an increasing focus on efficiency, health economics expertise is a key prerequisite for ensuring high-quality and economically viable care (2).

International developments show that health economics content has already been systematically and mandatorily implemented in numerous curricula (e.g., in the UK, the US, and the Netherlands) to prepare future physicians for these challenges. In contrast, there is currently no standardized curriculum or mandatory course offering for health economics in Germany. This structural deficit is problematic because economic aspects permeate all levels of care—from prevention and diagnosis to treatment and aftercare—and thus have a direct impact on clinical decision-making processes. Many students only recognize the relevance of this topic in later stages of their training, if at all, which leads to a systematic “curriculum gap.” This potentially impairs the decision-making and action-taking skills of young doctors [see (3, 4)].

In addition, the implementation of innovative teaching methods in Germany has so far met with considerable structural and cultural resistance. This is particularly true for digital teaching formats, which offer special didactic potential for complex, interdisciplinary content such as health economics. A precise conceptual differentiation seems necessary here: While health economics as a scientific discipline encompasses the analysis of economic processes in the healthcare system, the term “digital health economics education” refers to the methodological teaching of this content via digital formats.

The coronavirus pandemic (SARS-CoV-2) has not only exposed the structural deficits of the German healthcare system but has also permanently reinforced the need for health economics skills in the medical profession [see (5, 6)]. At the same time, the pandemic-driven digitization of teaching has demonstrated the relevance and potential of digital education formats for effectively and scalably conveying complex cross-disciplinary content.

Against this backdrop, the present study aims to assess the attitudes of medical students in Germany toward the introduction of a mandatory health economics teaching module. The focus is on the extent to which a need for health economics competence development is perceived and the degree to which digital teaching formats are accepted as a forward-looking teaching strategy.

## Materials and methods

### Study design and setting

This study was conducted as a prospective, cross-sectional pilot study to assess medical students’ attitudes toward the integration of health economics content into medical education. The cross-sectional design was deliberately chosen because this method is particularly well suited to capturing opinions and perceptions at a specific point in time without the need for longitudinal observation.

The study was conducted and reported in accordance with the STROBE recommendations (Strengthening the Reporting of Observational Studies in Epidemiology) (7).

### Ethics

The study protocol was reviewed and approved by the Ethics Committee of the University Hospital Jena (registration number: 2019-1456-Bef). Participation was voluntary and anonymous. All participants gave their electronic consent before the survey began.

### Participants and recruitment

**FIGURE 1:**
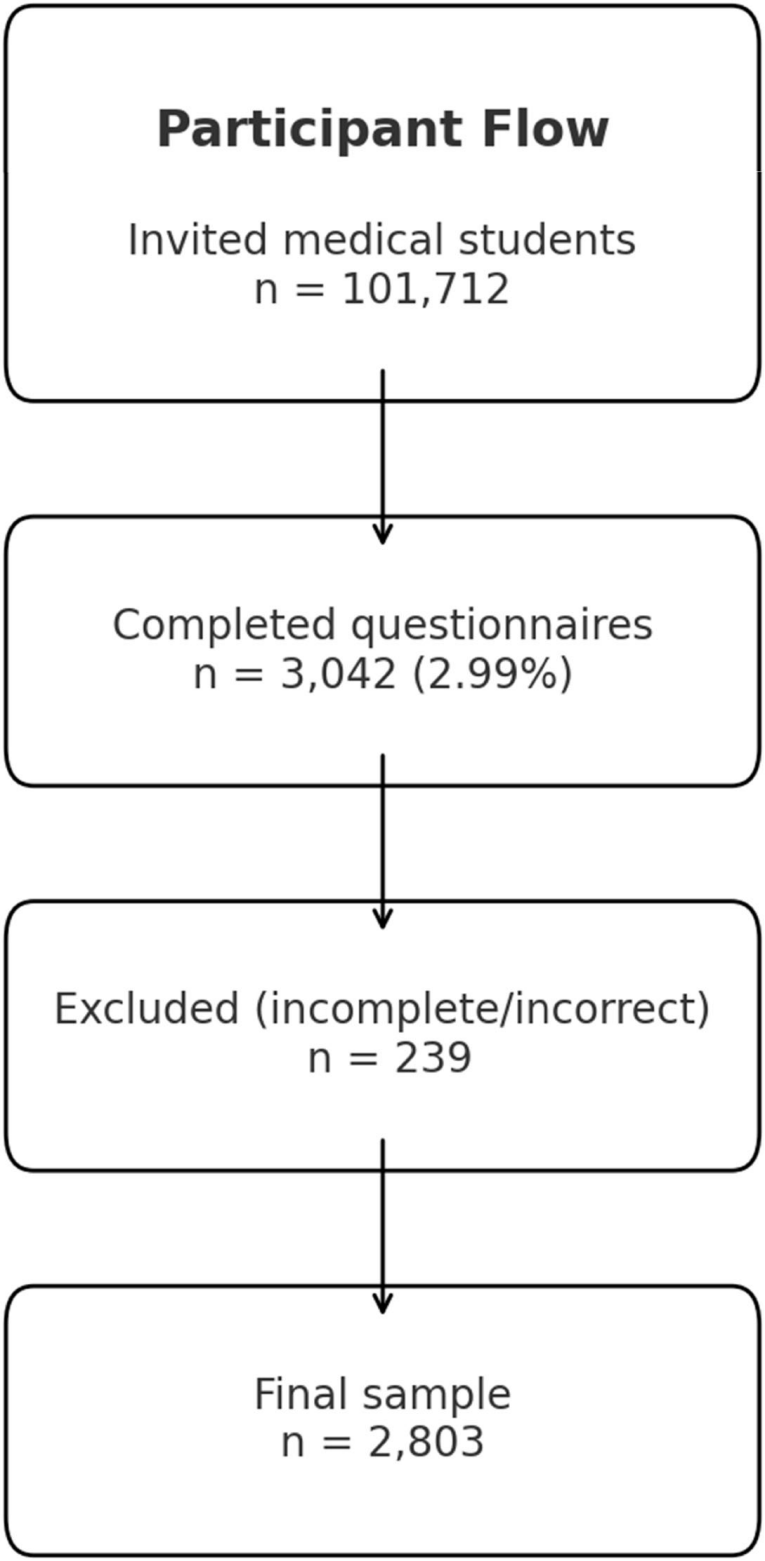
Participant flow.

The target group was all medical students at German medical faculties in the 2020/2021 academic year (*n* = 101,712) (8). The invitation to participate was sent via the official email distribution lists of the student councils at all 36 faculties in Germany. The online survey was available from April to October 2021. The inclusion criteria were: (1) age ≥ 18 years and (2) enrollment in human medicine at a German faculty. Questionnaires with missing information or incorrect multiple answers were excluded (Figure 1).

### Questionnaire development

The questionnaire was developed in close collaboration with the Chair of Health Economics at Friedrich-Alexander University Erlangen. It was based on published recommendations for online surveys (9–12) and comparable studies. Drafts were reviewed in several pre-tests within the research team. In addition, a pre-test was conducted with 20 randomly selected medical students to ensure comprehensibility and content validity. The final questionnaire was implemented using the SurveyMonkey tool (San Mateo, CA).

It comprised 24 questions in four thematic blocks.

- Sociodemographic information,
- Attitudes toward health economics.
- Assessments of the introduction of a compulsory module on health economics.

### Barriers and acceptance of digital teaching formats

The closed questions were answered on a five-point Likert scale (1 = strongly agree to 5 = strongly disagree). In addition, open-ended questions were asked to allow for free-text responses. The completion time was approximately 15 min.

### Handling missing data

To ensure data quality, only fully completed questionnaires were included in the analysis. Missing answers were not imputed.

### Data

The data was analyzed using SurveyMonkey and SPSS (version 17.0, SPSS Inc., Chicago, IL, USA).

Due to the expected non-normal distribution, non-parametric methods were used.

- Mann-Whitney U test for ordinal Likert data,
- Chi-square test for categorical variables, and
- Binomial test for dichotomous items.

The open-ended responses were evaluated using inductive, qualitative content analysis. Two independent coders performed the coding, with discrepancies resolved through a consensus process. The frequencies of central topic categories were also presented quantitatively.

### Sample

Of the 3,042 questionnaires received (response rate: 2.99%), 239 were excluded due to incomplete or incorrect information. This left 2,803 complete data sets for analysis.

### Bias and limitations

There is a potential self-selection bias, as students with a particular interest in health economics issues may have been more likely to participate in the survey. This may lead to an overestimation of positive attitudes. Furthermore, due to the relatively low response rate, the results can only be applied to the population of medical students to a limited extent (limited external validity).

Standardized, pre-tested questionnaires were used to minimize measurement errors.

## Results

### Epidemiological data from medical students

A total of 2,803 medical students took part. This corresponds to 2.76% of the 101,712 medical students who were enrolled at German medical faculties in the 2020/21 winter semester according to publicly available data from the Federal Statistical Office [Statistisches Bundesamt (13)]. A total of 1,947 women (69.4%) and 851 men (30.5%) took part. Five students (0.1%) identified as “diverse”. Of the responses to the survey, 14% (*n* = 392) came from the pre-clinical phase, 52% (*n* = 1458) from the clinical phase and 28% (*n* = 785) from the practical year. 6% (*n* = 168) of participants were in the “Other” category (free semester, leave of absence, etc.). About the gender distribution in the various semesters, significantly more female students (*f* = 231, *m* = 161; *p* < 0.001) responded to the questionnaire in the pre-clinical year out of the 392 students, and more male students (*f* = 303, *m* = 482; *p* < 0.001) responded in the practical year out of the 785 students. In the clinical field, 204 male students and 1,254 female students out of a total of 1,458 students responded to the questionnaire (*p* < 0.001). In the evaluation, the gender “diverse” was indicated in a small number of cases (*n* = 5). The average age of the students was 24.63 years (see Tables 1, 2).

**TABLE 1:** Overview of epidemiological data with confidence intervals for demographic data.

Group	Sample size (*n*)	Proportion (%)	95% Confidence interval
Female students	1947	69.4%	67.7%–71.0%
Male students	851	30.5%	28.9%–32.2%
Diverse students	5	0.1%	0.0%–0.2%
Pre-clinical phase	392	14%	12.7%–15.2%
Clinical phase	1458	52%	50.1%–53.9%
Practical year	785	28%	26.2%–29.8%
Other category	168	6%	5.1%–6.9%

**TABLE 2:** Overview of epidemiological data.

Study phase	Female	Male	Significance (*p*-value)
Pre-clinical	231	161	<0.001
Clinical	1254	204	<0.001
Practical year	303	482	<0.001

### Attitudes of medical students toward health economics

**FIGURE 2:**
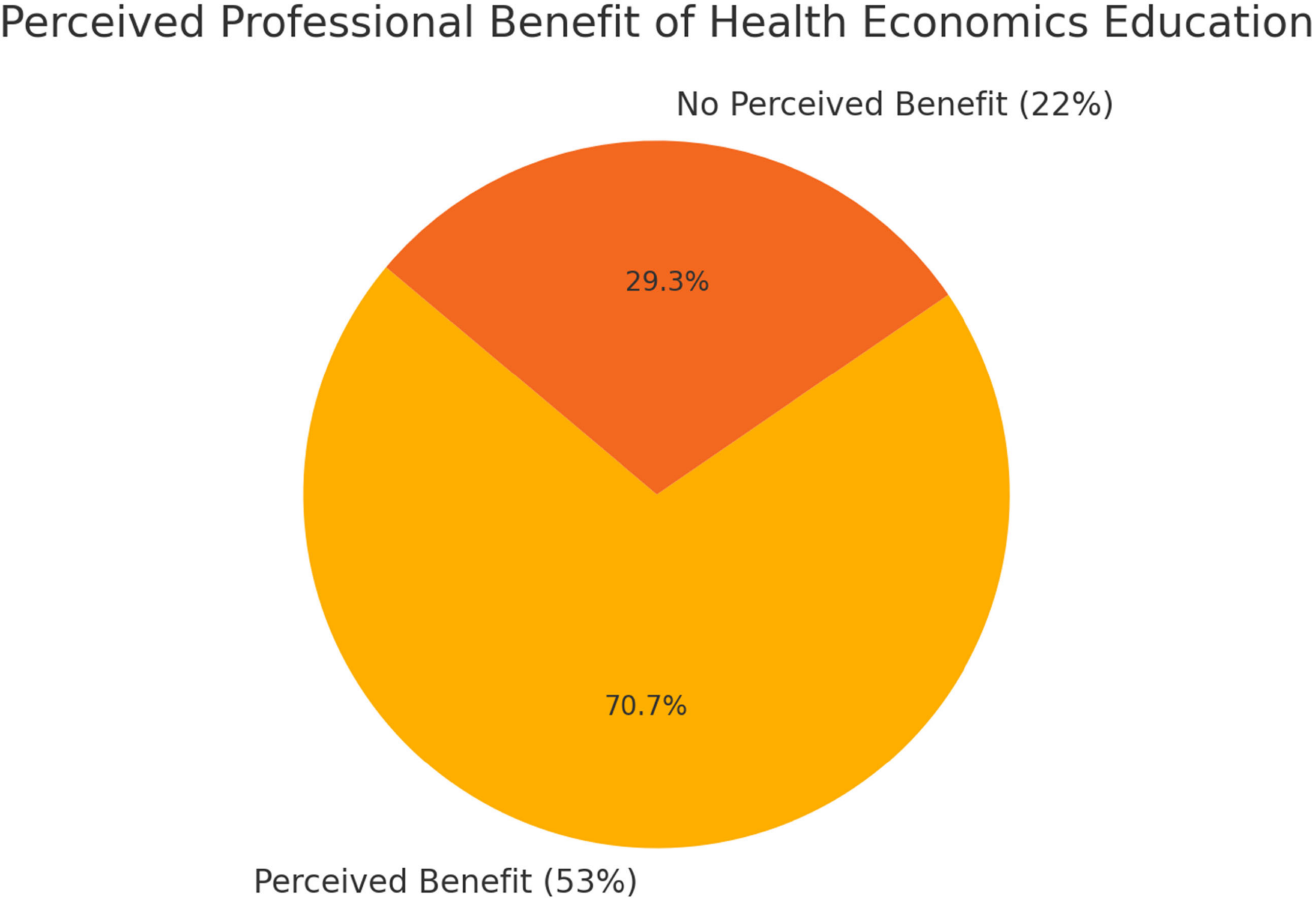
Benefit of health economics education.

A survey of 2,803 medical students was conducted to gather their attitudes and opinions on health economics in medical studies. Only 1% of respondents (*n* = 39) stated that they had already received health economics content in their studies, while 99% (*n* = 2,764) said they had not. The self-assessment of knowledge in this area was predominantly critical: 75% of students (*n* = 2,106) rated their knowledge as poor or at best satisfactory. In contrast, only 25% (*n* = 697) rated their knowledge as good to very good (Figure 2).

**FIGURE 3:**
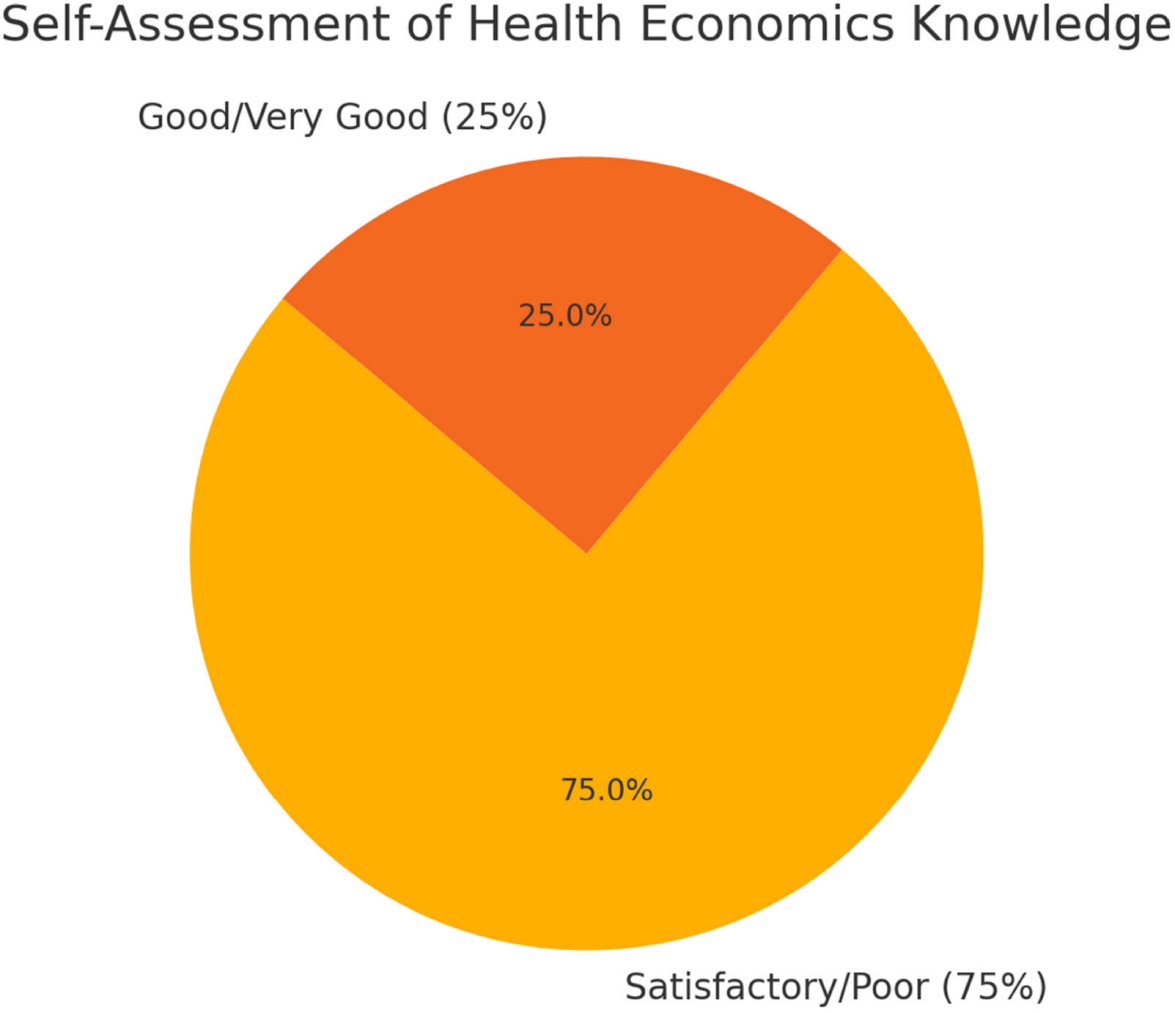
Self-assessment of health economics knowledge.

Most students consider health economics to be very important in medical education. For example, 76% (*n* = 2,124) considered the integration of relevant content to be important. Around 24% of respondents, however, see no added value (see Figure 3).

**FIGURE 4:**
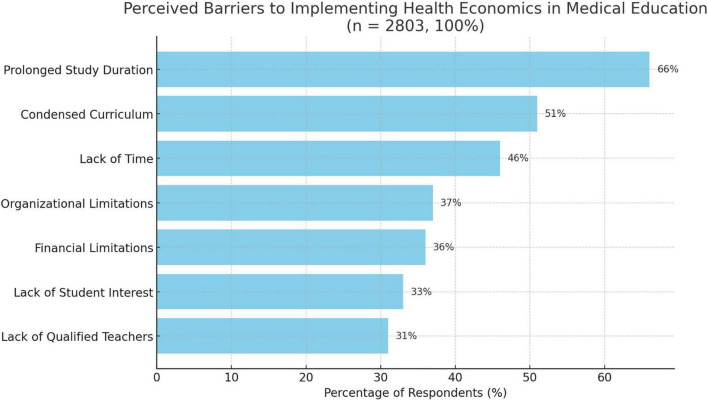
Barriers to implementation.

Opinions were mixed regarding the integration of health economics into the curriculum: 51% (*n* = 1,424) were in favor of integration into the compulsory curriculum, while 35% (*n* = 981) rejected this; 14% (*n* = 393) were neutral (see Figure 4).

**FIGURE 5:**
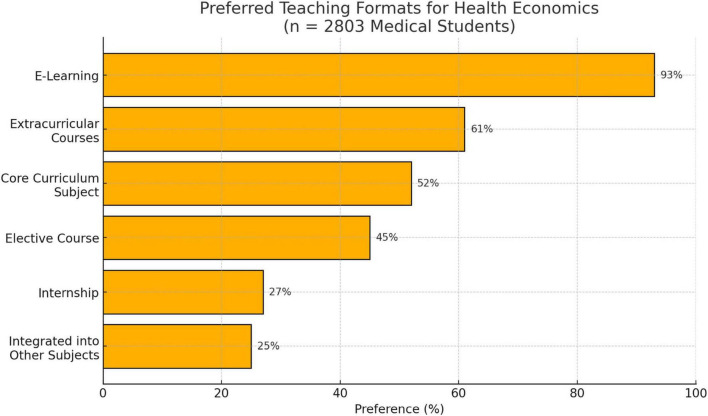
preferred teaching formats.

In addition, 53% (*n* = 1,495) of students stated that, in their view, the introduction of a teaching format on health economics could contribute to better preparation for their medical career. In contrast, 22% (*n* = 624) denied any such benefit (see Figure 5).

The students identified a possible extension of the duration of their studies (66%) and a condensation of the existing curriculum (51%) as particular obstacles to the implementation of health economics courses. Other challenges mentioned were lack of time (46%), lack of organizational (37%) and financial resources (36%), lack of interest among students (33%) and a shortage of qualified teaching staff (31%).

There was a high degree of openness regarding suitable teaching formats: 93% of respondents could imagine completing health economics content as part of an e-learning programme. 61% preferred extracurricular integration, while 52% were in favor of integration into the compulsory curriculum. Forty-five percent of students (*n* = 1,261) were in favor of implementing health economics as an elective subject. Twenty-five percent supported the integration of health economics content into existing subjects, while 27% (*n* = 757) considered an internship to be a useful format for the subject (see Figures 4, 5).

### Statistically significant preferences for health economics teaching formats E-learning as the preferred teaching format

The survey results show that medical students have a strong preference for e-learning as a format for teaching health economics content. Overall, 93% of respondents said they could imagine using an online format for this purpose. This result differs statistically significantly from all other formats surveyed.

To analyze the distribution of preferences in more detail, paired *z*-tests for independent samples were carried out. The preferences for e-learning were compared with those for other teaching formats. All tests revealed highly significant differences (*p* < 0.001). The greatest differences were found in the comparison between e-learning and the following formats (see Table 3).

**TABLE 3:** Overview of significant preferences for health economics teaching formats E-learning as the preferred teaching format.

Comparison	Proportions	*p*-Value	95% Confidence interval
E-learning vs. integration in other subjects	93% vs. 25%	<0.001	[0.661; 0.699]
E-learning vs. practical training	93% vs. 27%	<0.001	[0.641; 0.679]
E-learning vs. elective course	93% vs. 45%	<0.001	[0.459; 0.501]
E-learning vs. core curriculum	93% vs. 52%	<0.001	[0.389; 0.431]
E-learning vs. extracurricular	93% vs. 61%	<0.001	[0.300; 0.340]

### Influence of socio-demographic characteristics on e-learning in health economics

The analysis examined whether certain sociodemographic characteristics have an influence on acceptance of e-learning in health economics. No significant difference in acceptance was found for the characteristic of gender. The proportion of women who agreed was 92.0%, while that of men was 89.9% (*p* = 0.08199). A significant difference was found for the characteristic “stage of study” (*p* = 0.00004). The characteristic “age” also showed a significant difference in acceptance of e-learning (*p* = 0.00000). Younger students (<25 years) show significantly higher acceptance (see Table 4).

**TABLE 4:** Influence of socio-demographic characteristics on e-learning in the field of health economics.

Hypothesis	Female agreement (%)	Male agreement (%)	*p*-value (Chi^2^ test)	Significance
Gender influences E-learning preference	92.0	89.9	0.08199	Not significant
Study phase influences E-learning preference	–	–	0.00004	Significant
Age influences E-learning preference	–	–	0.0	Significant

### Detailed analysis of the seven barriers

An exploratory factor analysis of the perceived barriers to implementing health economics teaching revealed two main components. The first factor, labeled “structural constraints,” included the variables “compressed curriculum,” “lack of time,” and “long duration of study.” This suggests that these issues are related to curriculum overload and academic pressure. The second factor, referred to as “resource and motivation constraints,” included “lack of student interest,” “lack of qualified teaching staff,” “organizational constraints,” and “financial resources.” This points to a group of institutional and motivational barriers. To this end, a logistic regression analysis was conducted to examine predictors of the acceptance of e-learning formats in health economics education. Of the seven predictors, only financial constraints showed a statistically significant negative association with the acceptance of e-learning (*p* = 0.027). This suggests that concerns about financial resources may inhibit support for digital teaching formats. The remaining variables—such as the compressed curriculum, lack of time, student interest, availability of teaching staff, organizational capacities, and extended duration of study—were not statistically significant. The regression model, based on a sample of 2,803 observations, yielded a pseudo R^2^-value of 0.003, indicating limited explanatory power. Additional regression analyses revealed significant correlations between age and stage of study on the one hand and students’ preference for e-learning in health economics on the other (both *p* < 0.001). Younger students and students in the pre-clinical phase showed a higher level of agreement with digital formats.

## Discussion

Health economics has played a minor role in medical education at German universities to date, although it is becoming increasingly important in view of the growing economization of the healthcare system (14). The present study confirms that most medical students consider their knowledge in this area to be inadequate and at the same time express a keen interest in health economics teaching.

Despite an increasing number of health economics courses (15), there is a significant discrepancy between economic management in everyday clinical practice and medical education. Economic decision-making processes, e.g., through the DRG system, are increasingly influencing medical practice, which is viewed critically by doctors (16). At the same time, prospective doctors lack the in-depth knowledge to be able to competently shape these processes (17).

However, students fear that additional content will overwhelm them (18). This concern is justified, as an increasingly dense curriculum is associated with stress and psychological strain, which can have a negative impact on academic success and health (19).

The results also show that digital formats such as e-learning are rated particularly positively, especially by younger and pre-clinical students. This confirms current findings on the high acceptance of digital teaching offerings in medical education (20).

Early and digitally supported access to health economics can help to avoid existing prejudices and build skills that are necessary for interdisciplinary collaboration with economists (21). Corresponding initiatives, e.g., at the University of Tübingen, demonstrate the potential of such models.

This study examines medical students’ attitudes toward health economics teaching and their preferred teaching formats, with a particular focus on digital offerings. The results show a high level of approval for digital teaching formats: 93% of students are in favor of e-learning for health economics. This result is in line with current trends toward the digitization of medical education, which demonstrate the increasing acceptance and effectiveness of digital formats (21).

The analysis showed that gender has no significant influence on the approval of e-learning. Women and men were almost equally supportive of digital formats. However, significant differences were found for the stage of study and the age of the students. Students in the pre-clinical phase and under the age of 25 showed higher levels of approval. These results can be explained by different requirements and learning habits in the various phases of training. Previous studies show that younger students and students in earlier phases of their studies tend to be more open to digital formats (19).

The high level of acceptance of digital health economics teaching highlights the potential for systematic integration of e-learning modules into medical studies. To date, medical education has often lacked health economics content embedded in the curriculum (14, 16, 20), even though basic economic knowledge is becoming increasingly important for prospective doctors. Digital formats could provide flexible and scalable access to close this gap.

The increasing economization of the healthcare system poses new challenges for medical education. Health economics issues are increasingly shaping medical practice, yet there is often a lack of systematic teaching in this area in medical studies (14, 18). The results of this study show that many medical students consider their knowledge to be insufficient, but at the same time there is a great deal of interest in health economics content.

An innovative approach to teaching this content lies in “digital health economics education.” This newly defined term describes digitally supported health economics teaching in medical studies and combines economic knowledge with modern, flexible teaching methods. Digital formats such as e-learning are supported by an overwhelming majority (93%) of students – especially younger and pre-clinical students, which is consistent with previous findings on digital affinity in early stages of education (19).

Digital health economics education (DHEE) can be seen as a building block in a multidimensional solution to strengthen economic skills in the medical profession. In view of the profound structural challenges facing the German healthcare system, such as the economic difficulties of many hospitals and health insurance companies, the integration of such content is becoming increasingly urgent. The discussion about economic decision-making constraints in everyday hospital life, e.g., due to the DRG system, requires a sound understanding to be able to weigh up economic efficiency and medical ethics (21).

The implementation of digital health economics teaching modules can help to break down existing barriers, prevent prejudices and promote interdisciplinary skills (21). In the future, DHEE should become an integral part of the curriculum at medical schools. However, anchoring it in the curriculum requires close cooperation between medical professionals and health economists, both in theory and in practice. In the long term, this could develop into an independent branch of education that contributes to the professionalization of interdisciplinary health education (22, 23).

Digital health economics education is therefore not only an innovative teaching format, but also a forward-looking response to the structural transformation processes in the healthcare system. It addresses both the needs of learners and the systemic requirements of an economically driven healthcare reality (24).

### Systemic integration and interoperability of digital modules

The successful implementation of digital health economics training formats requires not only a conceptual realignment, but also structural measures at the university level. Of relevance here is the question of interoperability between different faculties, study locations, and learning platforms. To ensure the comparability and recognition of such teaching formats within the framework of uniform national standards, the development of a cross-university curriculum should be considered. This could be based on modular ECTS-based units that are recognized across faculties and, if necessary, offered via central platforms such as those offered by (25).

### Specific examples of existing digital teaching formats in Germany

Some universities have already initiated innovative projects for digital teaching in the field of medicine. Relevant aspects include, among others: digital knowledge portals, interactive learning platforms, digital modules for medical decision-making integrated into learning objective-oriented curricula and blended learning formats with an economic focus have been piloted, for example in the context of public health or healthcare management.

These initiatives demonstrate that both the technological infrastructure and the didactic potential already exist. However, there needs to be political and institutional willingness to systematically and bindingly integrate the digital health economy into medical training (26–28).

## Limitations

This study has several methodological limitations that should be considered when interpreting the results. First, it should be noted that the low response rate (2.99%) could indicate self-selection among participants. Students who have a fundamental interest in health economics topics may have been more willing to participate. This can lead to a response bias, which limits the external validity of the results. Furthermore, the data collection is based on self-reported information, which is potentially subjective and may lead to socially desirable responses.

Another methodological aspect concerns the cross-sectional survey, which only provides a snapshot of students’ attitudes at a given point in time. No conclusions about causal relationships or temporal developments can be drawn from the available data.

### Future research directions

To further develop this field of research, future studies should use controlled study designs, such as randomized controlled trials (RCTs), to systematically investigate the effectiveness of digital teaching formats in the field of health economics (DHEE). It is possible to measure both the learning gains and the competence development of medical students longitudinally. In addition, students’ attitudes and behavioral changes could also be examined. The use of qualitative studies, such as interviews with teachers or curriculum managers, could provide additional insights into institutional and cultural barriers.

In the long term, outcome-oriented studies would also be desirable to investigate the practical effects of DHEE on medical decision-making, resource management, and interdisciplinary collaboration in everyday clinical practice—for example, by comparing cohorts with and without DHEE training.

## Conclusion

### For decision-makers in higher education policy

The results of the study underscore the relevance of strategically integrating health economics content into medical studies, with digitally supported teaching formats considered advantageous. The implementation of DHEE modules in national competency catalogs and accreditation standards could contribute to standardization and quality assurance.

### For medical faculties and curriculum developers

The high acceptance of digital formats, especially among preclinical and younger students, highlights the need for targeted curricular integration of digital health economics modules. Faculties should critically examine existing reservations about digital formats and explore cooperation models with health economics institutes and digital education providers.

### For teachers and lecturers

The results of the study suggest that students have a high affinity for digital formats, if they are designed in a didactically meaningful and practice-oriented manner. Lecturers should therefore specifically expand their digital teaching skills and embrace new formats such as blended learning or microlearning.

### For students

This study emphasizes the growing interest in and relevance of health economics knowledge for future medical practice. It is recommended that students actively use and demand relevant courses, especially in the digital field, to prepare themselves as well as possible for the complex challenges of an economized healthcare system.

In summary, it can be said that DHEE – in the sense of DHEE – is widely accepted by medical students and is particularly well received by younger and pre-clinical students. The results provide important insights for the further development of medical curricula in the digital age.

## Data Availability

The original contributions presented in this study are included in this article/supplementary material, further inquiries can be directed to the corresponding author.
